# PET-CT detection of local residual laryngeal carcinoma after definitive (chemo)radiotherapy

**DOI:** 10.1186/s12885-023-10834-1

**Published:** 2023-04-18

**Authors:** Heli J. Sistonen, Taru Ilmarinen, Timo Atula, Katri Aro, Jukka Schildt, Antti Markkola

**Affiliations:** 1 Department of Radiology, University of Helsinki and Helsinki University Hospital, Haartmaninkatu 4, Helsinki, 00029 HUS Finland; 2 Department of Otorhinolaryngology – Head and Neck Surgery, University of Helsinki and Helsinki University Hospital, Kasarmikatu 11–13, Helsinki, 00029 HUS Finland; 3 Department of Nuclear Medicine, University of Helsinki and Helsinki University Hospital, Haartmaninkatu 4, Helsinki, 00029 HUS Finland

**Keywords:** Positron emission tomography and computed tomography, Post-treatment, Chemoradiotherapy, Imaging, Head and neck cancer

## Abstract

**Background:**

Positron emission tomography and computed tomography (PET-CT) is currently recommended in evaluating the treatment response after (chemo)radiotherapy ([C]RT). In the larynx, post-treatment changes and physiological uptake make image interpretation more challenging compared to other head and neck sites. Previous research has not addressed imaging factors specifically in the larynx that would help in distinguishing the residual disease and explain the unique challenges of that anatomic area. The study cohorts are small and heterogenous. Our objective was to investigate the ability of PET-CT in diagnosing local residual laryngeal carcinoma, and to uncover imaging factors that could be used in differentiating the residual disease from post-treatment and physiological changes. In the same study cohort, we also aimed to uncover prognostic factors for local residual or recurrent disease.

**Methods:**

Our retrospective study cohort included 73 patients with T2-T4 laryngeal carcinoma undergoing (C)RT with curative intention, and post-treatment non-contrast-enhanced PET-CT at 2–6 months. Findings were compared between local residual and non-residual disease. Local residual disease was defined as a persistent tumor growth with no evidence of remission in between, confirmed by biopsy, and evident within 6 months from the end of RT. PET-CT was evaluated using a 3-step scale: negative, equivocal, and positive.

**Results:**

Nine (12%) had a local residual tumor and 11 (15%) developed local recurrence, based on the biopsy. The median follow-up of surviving patients was 64 months (range, 28–174). In univariate analysis, primary tumor diameter greater than 2.4 cm (median value), and vocal cord fixation were prognostic for local residual or recurrent disease. Sensitivity, specificity, PPV, and NPV were 100%, 75%, 36%, and 100%, respectively, when the equivocal interpretation was grouped with the positive interpretation. All local residuals, and 28% (18/64) non-residuals, had a primary tumor area SUV_max_ of over 4.0 (p < 0.001). CT showed a persistent mass at the primary tumor area in 56% of residuals, and in 23% of non-residuals (p > 0.05). By combining SUV_max_>4.0 and mass, specificity improved to 91%.

**Conclusions:**

NPV of post-treatment PET-CT in laryngeal carcinoma is high, but equivocal and positive results have low PPV and require further diagnostics. All local residuals had SUV_max_ over 4.0. The combination of SUV_max_ over 4.0 and mass on CT increased specificity, but the sensitivity was low.

## Background

Radiotherapy (RT) and chemoradiotherapy (CRT) are important function-preserving treatment options for laryngeal carcinoma [1]. After surgery, margins are evaluated to determine the complete tumor resection. After (C)RT, however, the treatment response evaluation is based on endoscopy and imaging. Endoscopically, the detection of residual or recurrent laryngeal carcinoma can be difficult due to post-treatment mucosal edema. Growth is often multicentric and covered by an intact mucosa [2], and direct laryngoscopy and biopsies often fail to detect the tumor [3, 4]. After definitive (C)RT, laryngeal carcinoma should be screened with PET-CT at 3 months [5–7]. According to a review and meta-analysis in 2016, for detecting a local residual or recurrent head and neck cancer (HNC), the pooled estimates of sensitivity, specificity, positive predictive value (PPV), and negative predictive value (NPV) were 86.2%, 82.3%, 52.7% and 96.3%, respectively [8]. For laryngeal carcinoma, according to another review, the corresponding figures were 33–75%, 53–86%, 40–67% and 73–86% [5]. Thus, PPV in HNC is low, but interpreting post-treatment PET-CT can be even more challenging in laryngeal carcinoma. Physiologic uptake and post-radiation edema, mucositis and radionecrosis increase false positive findings [9], and small tumor foci may be difficult to detect, leading to suboptimal sensitivity. To our knowledge, there are no studies that aim to solve these problems and improve interpretation specifically in laryngeal carcinoma.

Our primary aims are to investigate the ability of post-treatment PET-CT in uncovering residual disease within the larynx, and to document the spectrum of imaging findings so that they could be used more reliably in the treatment response evaluation. Our secondary aim was to uncover pre-treatment patient or imaging characteristics that are prognostic for local residual or recurrent disease.

## Materials and methods

### Patients

For this retrospective study we included patients with T2-T4 laryngeal carcinoma, diagnosed at Helsinki University Hospital Department of Otorhinolaryngology – Head and Neck Surgery between 2006 and 2018, and who underwent (C)RT with curative intention, and had post-treatment PET-CT examination at 2–6 months. The patients were retrieved from the hospital database using the International Classification of Diseases code C32. We excluded patients with previous HNC. Further, two patients were excluded because the maximum standard uptake values (SUV_max_) could not be retrospectively measured due to technical difficulties. The final study cohort consisted of 73 patients, including 71 squamous cell carcinomas, 1 adenocystic carcinoma, and 1 sarcomatotic carcinoma. Table 1 includes patient demographics, pre-treatment imaging findings, and survival data, stratified by primary area residual or recurrent disease.

**Table 1:** Patient demographics, treatment results and pre-treatment imaging findings

	All patients, n = 73	Residual or recurrent disease, n = 20	No residual or recurrent disease, n = 53	p-value
Gender, (%)				0.329
Female	15 (20.5)	6 (30.0)	9 (17.0)	
Male	58 (79.5)	14 (70.0)	44 (83.0)	
T-stage, (%)				0.128
T1	0 (0)	0 (0)	0 (0)	
T2	28 (38.4)	4 (20.0)	24 (45.3)	
T3	36 (49.3)	13 (65.0)	23 (43.4)	
T4a	8 (11.0)	2 (10.0)	6 (11.3)	
T4b	0 (0)	0 (0)	0 (0)	
Missing	1 (1.4)	1 (5.0)	0 (0)	
N-stage, (%)				0.111
N0	54 (74.0)	17 (85.0)	37 (69.8)	
N1	7 (9.6)	0 (0)	7 (13.2)	
N2a	1 (1.4)	1 (5.0)	0 (0)	
N2b	4 (5.5)	0 (0)	4 (7.5)	
N2c	6 (8.2)	1 (5.0)	5 (9.4)	
N3	0 (0)	0 (0)	0 (0)	
Missing	1 (1.4)	1 (5.0)	0 (0)	
N-stage, (%)				0.241
N0	54 (74.0)	17 (85.0)	37 (69.8)	
N1-N2x	19 (26.0)	3 (15.0)	16 (30.2)	
Grade, (%)				0.587
G1	9 (12.3)	1 (5.0)	8 (15.1)	
G2	44 (60.3)	13 (65.0)	31 (58.5)	
G3	12 (16.4)	4 (20.0)	8 (15.1)	
Missing	8 (11.0)	2 (10.0)	6 (11.3)	
Smoking at diagnosis, (%)				0.187
Yes	50 (68.5)	12 (60.0)	38 (71.7)	
Quit earlier	16 (21.9)	4 (20.0)	12 (22.6)	
Never	7 (9.6)	4 (20.0)	3 (5.7)	
Location of primary tumor according to the ICD code, (%)				0.176
C32.0 Glottic	29 (39.7)	8 (40.0)	21 (39.6)	
C32.1 Supraglottic	20 (27.4)	4 (20.0)	16 (30.2)	
C32.2 Subglottic	2 (2.7)	2 (10.0)	0 (0)	
C32.8 Overlapping sites	22 (30.1)	6 (30.0)	16 (30.2)	
Treatment, (%)				0.742
Chemoradiotherapy	60 (82.2)	16 (80.0)	44 (83.0)	
Radiotherapy only	13 (17.8)	4 (20.0)	9 (17.0)	
Cartilage erosion, (%)				1.000
Yes	28 (38.4)	8 (40.0)	20 (37.7)	
No	45 (61.6)	12 (60.0)	33 (62.3)	
Paraglottic extension, (%)				0.551
Yes	55 (75.3)	14 (70.0)	41 (77.4)	
No	18 (24.7)	6 (30.0)	12 (22.6)	
Died of laryngeal cancer, (%)				**0.012**
Yes	17 (23.3)	9 (45.0)	8 (15.1)	
No	56 (76.7)	11 (55.0)	45 (84.9)	
Second primary, (%)				0.763
Yes	18 (24.7)	4 (20.0)	14 (26.4)	
No	55 (75.3)	16 (80.0)	39 (73.6)	

The data is stratified by the presence of residual or recurrent disease **at the primary tumor area** (verified by biopsy specimen)

Multidisciplinary tumor board meetings reviewed the diagnostics and staging for all patients and gave treatment recommendations according to national guidelines. Altogether, 60 (82%) patients were treated with CRT and 13 (18%) with RT alone. The mean RT dose was 69.6 Gy (range 60–72). The majority of patients received Cisplatin 40 mg/m^2^, which was administered weekly with a maximum of 6 doses (median 5; range 1–6). The follow-up time frame for patients ranged from 28 to 174 months (median, 64 months), or until death. Of all 35 surviving patients at the end of our evaluation period, 33 (94.3%) had a minimum follow-up of 3 years.

Local remission was defined as imaging without any evidence of disease, or negative biopsies from the primary tumor area. Definition for local residual disease was a persistent tumor with no proof of remission, confirmed with biopsy, and evident within 6 months from the end of radiotherapy. Local recurrence was defined by a new tumor growth confirmed with biopsy after the patient’s previous remission.

### Imaging

Pre-treatment scans were retrospectively re-evaluated by a radiologist (H.S.) specialized in head and neck imaging. Altogether, 44 patients had MRI, 25 had CT, and 1 had PET-CT. One patient had both CT and MRI, one patient had MRI and PET-CT and one patient had CT and PET-CT.

After treatment, the patients were followed up with regular clinical examinations and PET-CT at 2–6 months. The median time from the end of treatment to the first follow-up PET-CT was 94 days (range 68–176 days). Post-treatment PET-CT images were retrospectively re-evaluated by a radiologist specialized in head and neck imaging (H.S.) and briefed to the PET-CT interpretation by a nuclear medicine specialist (J.S.). The radiologist was blinded to the original report and to the results of treatment. When the interpretation differed from the original report assessed by a nuclear medicine physician and a radiologist, a consensus was made together with another head and neck radiologist (A.M.).

Similarly to the original reporting style, imaging studies were re-assessed using a 3-step scale: negative for malignant disease, equivocal, and positive for malignant disease. The interpretation was negative when there was no abnormal uptake or only minimal mucosal linear uptake present. The interpretation was equivocal when focal mild uptake or moderate mucosal linear uptake was observed. The interpretation was positive when there was intense focal uptake, enlarging or a new mass. SUV_max_ of the primary tumor area and abnormal activity in the larynx and neck lymph node areas were measured. No particular SUV_max_ value was used to distinguish between malignant and benign tissue. SUV_max_ in abnormal uptake areas and on the primary tumor area were measured with an automatic 3D tool or when that was not possible, by drawing a region of interest (ROI), encompassing the whole area with the abnormal uptake. In addition, findings were classified according to the NI-RADS qualitative criteria [10]. The software used to analyze PET-CT scans included Hermes (Hermes Medical Solutions, Stockholm, Sweden) and Syngo.via (Siemens Healthcare GmbH, Erlangen, Germany).

The patients fasted for six hours and had blood glucose levels < 10 mmols before the imaging. After an intravenous injection of approximately 350–450 MBq (5 MBq/kg) of 18-Fluoride-fluorodeoxyglucose (18 F-FDG) and 60 min of resting, images were acquired on an integrated PET-CT scanner. Body images were obtained separately from head and neck images. CT was imaged from the jugulum to mid-thigh, with the patients’ arms raised above shoulder level. CT parameters were 120–140 kV, 50–60 mAs and a section width was 4 mm. The examination was followed by a PET scan, with an 8 cm bed position and 1.5 min per frame. The head and neck were imaged with the patients’ arms positioned down. CT parameters were 120 KeV, 50 mAs, and a section width of 3 mm. PET had an 8 cm bed position and 2.5 min per frame. CT was performed without contrast, per the institutional protocol. The PET-CT system was Philips Gemini GXL (Philips Healthcare, Best, Netherlands) until 2015, and Siemens Biograph (Siemens Healthcare, IL, USA) thereafter. A correction factor was used to transform SUV measures to be comparable between the two systems.

### Statistical analysis

The times to recurrence and endpoints were counted from the last date of RT. Follow-up was defined as from the last date of RT to the last clinical visit. We calculated the sensitivity, specificity, PPV, NPV, and accuracy of PET-CT in assessing local residual tumors. Differences in categorical and ordinal variables were compared with a Chi-square test or Fisher’s exact test and in continuous variables with a Mann-Whitney-U nonparametric test. Cox logistic regression and Kaplan-Meier analysis were utilized to calculate association of pre-treatment factors with local residual or recurrent disease within 5 years. The primary tumor area SUV_max_ value for optimal sensitivity and specificity was assessed using receiver operating characteristic (ROC) curve analysis. A p-value of ≤ 0.05 was considered statistically significant.

For the statistical analysis, the PET-CT 3-step scale for residual disease was transformed into a two-class variable; we used both sensitive and specific interpretations. In the sensitive interpretation, the equivocal result was interpreted as positive, and in the specific interpretation, the equivocal result was interpreted as negative [11]. Although no specific SUV_max_ value was used in the assessment of malignancy, for statistical purposes, we selected SUV_max_>3.0 as an abnormal uptake-variable. The statistical analysis was performed with SPSS (IBM SPSS Statistics Version 27, Armonk, NY).

## Results

The patient and tumor data are presented in Table 1. The mean age of the patients was 61.9 years (range, 31–85 years). The mean pre-treatment tumor diameter was 2.7 cm (median, 2,4 cm, range, 1.3-7.8 cm), and the mean pre-treatment hemoglobin 141 g/l (median, 142 g/l, range, 104 g/l-170 g/l).

Of 73 patients 10 (14%) had residual disease: 9 in the area of primary tumor, and 1 in regional lymph nodes. The median time from the end of treatment to the diagnosis of primary tumor area residual disease was 4.3 months (range 2.3-6.0). Of 73 patients 12 (16%) had recurrent disease: 11 presented with local, and 1 with regional recurrence. Seven (9.6%) presented with distant metastases, of which 4 were discovered in the post-treatment PET-CT and 3 later during the follow-up. One of these also had local recurrence. Altogether, 18 out of all 20 patients with local residual or recurrent disease (90.0%) underwent salvage surgery; 9 who had a residual tumor, and 9 who had a recurrent tumor.

The larynx preservation rate (LPR, crude proportion) was 75.3%, and 3-year disease specific survival (DSS) was 83.6%. 3-year DSS in different T-stages was 92.9%, 77.8%, and 75.0%, in T2, T3, and T4a, respectively. DSS between different T-stage categories was not statistically different.

In the 5-year univariate Cox logistic regression analysis, pre-treatment vocal cord fixation and primary tumor diameter larger than median 2.4 cm were associated with local residual and recurrent disease. HR for vocal cord fixation was 2.649 (95% CI 1.002–7.004, p = 0.050), and HR for primary tumor diameter was 2.771 (95% CI 1.050–7.313, p = 0.039). T-stage 3–4 versus 2 was approaching significance (p = 0.064) for inferior local control. Multivariable analysis (adjusted for primary tumor size, vocal cord fixation, and T-stage [T2 versus T3-4]) showed no statistically significant prognostic factors.

### PET-CT

**Fig. 1:**
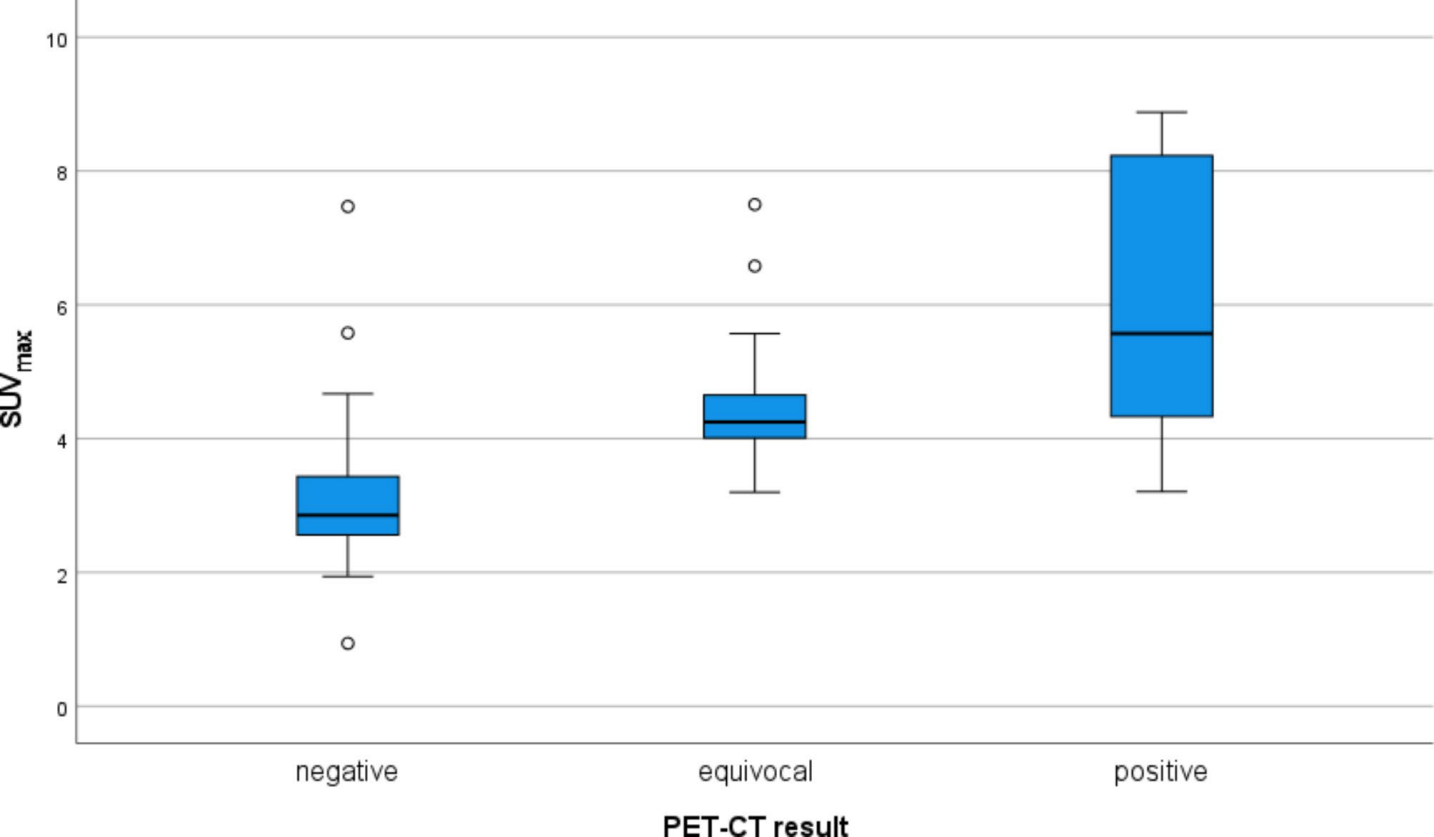
SUV_max_ in positive, equivocal and negative interpretation categories. The bars represent 25th and 75th percentiles, and whiskers represent minimum and maximum values, excluding outliers (circles)

Table 2 lists PET-CT variables stratified by the presence of primary tumor area residual disease, and Table 3 lists the sensitivity, specificity, PPV, NPV, and accuracy of different PET-CT parameters and reporting methods. On the primary tumor area, the PET-CT interpretation was negative in 48 of 73 patients (65.8%), and none had a residual tumor present. In 13 (17.3%) patients, of whom the interpretation was equivocal, 5 out of 13 (38.5%) had residual disease confirmed by a biopsy specimen. In 12 (16.4%) patients, of which the interpretation was positive, 4 out of 12 (33.3%) had residual disease. The mean SUV_max_ in negative, equivocal and positive interpretation categories were 3.097, 4.619 and 6.023 (p > 0.001), respectively (Fig. 1). Similarly, a mass was present on the CT in 14.6%, 15.4%, and 91.7%, respectively (p < 0.001) (Table 4).

**Table 2:** Post-treatment PET-CT results

Post-treatment follow-up PET-CT at 3 months	All patients n = 73	Residual n = 9	No residual n = 64	p-value
Abnormal uptake at primary tumor area, (%)*				**0.008**
Yes	42 (57.5)	9 (100)	33 (51.6)	
No	31 (42.5)	0 (0)	31 (48.4)	
Mass on primary tumor area, (%)				0.103
Yes	20 (27.4)	5 (55.6)	15 (23.4)	
No	53 (72.6)	4 (44.4)	49 (76.6)	
SUV_max_ over 4, (%)				**< 0.001**
Yes	27 (37.0)	9 (100.0)	18 (28.1)	
No	46 (63.0)	0 (0)	46 (71.9)	
SUV_max_ over 4 and mass on primary tumor area, (%)				**0.003**
Yes	11 (15.1)	5 (55.6)	6 (9.4)	
No	62 (84.9)	4 (44.4)	58 (90.6)	
Strong symmetric uptake, suggesting muscle activation, (%)				0.255
Yes	8 (11.0)	2 (22.2)	6 (9.4)	
No	65 (89.0)	7 (77.8)	58 (90.6)	

The data is stratified by the presence of residual disease **at the primary tumor area** (verified by biopsy specimen)

*abnormal uptake is defined as SUV_max_ over 3.0

**Table 3:** Sensitivity, specificity, positive predictive values, and negative predictive values of various PET-CT reporting methods

	Sensitivity, % (95%CI)	Specificity, % (95%CI)	PPV, % (95%CI)	NPV, % (95%CI)	Accuracy, %
Sensitive interpretation	100.0 (66.7–100.0)	75.0 (62.8–87.3)	36.0 (4.7–67.4)	100.0 (93.8–100.0)	78.1
Specific interpretation	44.4 (0-93.1)	87.5 (78.8–96.2)	33.3 (0-79.5)	91.8 (84.6–99.0)	82.2
NI-RADS	66.7 (29.0-100.0)	87.5 (78.8–96.2)	42.9 (3.3–82.5)	94.9 (89.1–100.0)	84.9
SUV_max_ over 3.0	100.0 (66.7–100.0)	48.4 (30.8–66.0)	21.4 (0-48.2)	100.0 (90.3–100.0)	54.8
SUV_max_ over 4.0	100.0 (66.7–100.0)	71.9 (58.9–84.9)	33.3 (2.5–64.1)	100.0 (93.5–100.0)	75.4
Mass on primary tumor area	55.6 (12.1–99.2)	76.6 (64.8–88.5)	25.0 (0–63.0)	92.5 (85.1–99.9)	74.0
SUV_max_ over 4.0 and mass on primary tumor area	55.6 (12.1–99.2)	90.6 (83.1–98.1)	45.5 (1.9–89.1)	93.5 (87.2–99.8)	86.3

In the sensitive interpretation, the equivocal result was interpreted as positive, and in the specific interpretation, the equivocal result was interpreted as negative

**Table 4:** SUV_max_ and mass in different interpretation categories

	Negative interpretation n = 48	Equivocal interpretation n = 13	Positive interpretation n = 12	p-value
SUV_max_				**< 0.001**
Mean	3.097	4.619	6.023	
Median	2.855	4.250	5.570	
Range	0.94–7.47	3.20–7.50	3.21–8.88	
Mass on CT, (%)				**< 0.001**
Yes	7 (14.6)	2 (15.4)	11 (91.7)	
No	41 (85.4)	11 (84.6)	1 (8.3)	

Of 8 patients, in whom the PET-CT was interpreted as equivocal and did not have residual disease, none developed later recurrence on the primary tumor area. Of 8 patients in whom the PET-CT was positive but did not have residual disease, 3 (37.5%) developed later recurrence on the primary tumor area. These three recurrences occurred at 7, 9, and 11 months after treatment. Findings in the post-treatment PET-CT could not predict future local recurrence.

In 16 (21.9%), the PET-CT interpretation locally was false positive, which included positive or equivocal interpretation, with negative biopsy results. There were no patient, treatment or imaging variables including the time point of post-treatment imaging that were statistically significantly associated with false positive interpretations compared to real residual disease.

The sensitivity, specificity, PPV, and NPV of the PET-CT in assessing local residual tumors did not differ significantly between patients scanned over 3 months after treatment compared with those scanned at 2–3 months after treatment.

### PET-CT findings characterization

**Fig. 2:**
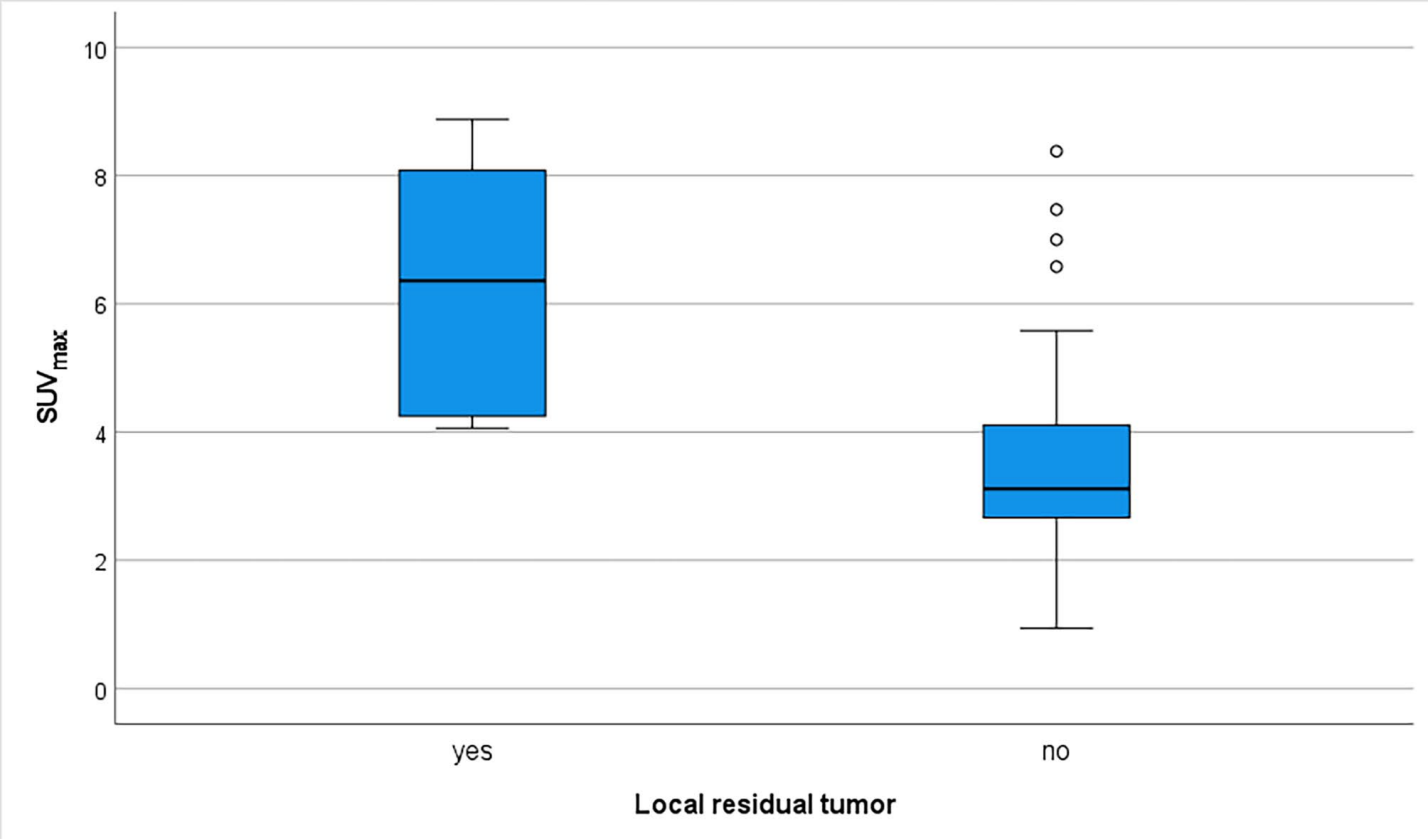
SUV_max_ in residuals and non-residuals. The bars represent 25th and 75th percentiles, and whiskers represent minimum and maximum values, excluding outliers (circles)

The mean and median SUV_max_ in primary tumor area residual disease were 6.267 and 6.360 (range 4.060–8.880), respectively, and in non-residuals, 3.508 and 3.115, respectively (range 0.940–8.380) (p < 0.001) (Fig. 2). All true local residual tumors had SUV_max_ over 4, but 11 of the 16 (68.8%) false positive interpretations had SUV_max_ over 4. Thus, the SUV_max_ values overlapped. The remaining mass on the primary tumor area existed in 5 out of 9 (56%) of the local residuals, and 15 out of 64 (23%) of the non-residuals (p > 0.05). When SUV_max_ over 4.0 was combined with residual mass, specificity and NPV rose to 91% and 94%, respectively. None had developed a new or increased mass on the primary tumor area.

**Fig. 3:**
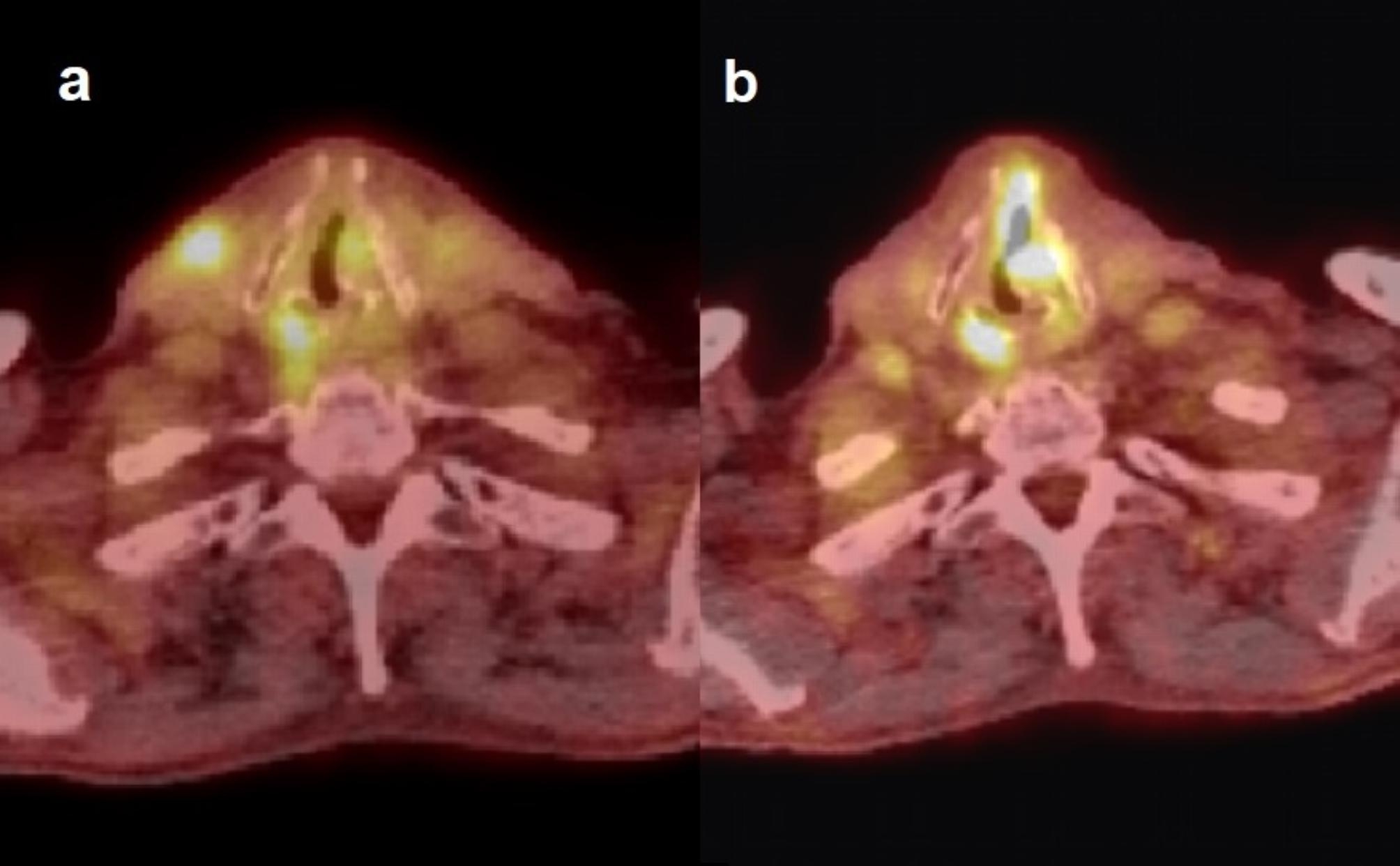
Possible false negative biopsy result after a positive PET-CT in a T3N0M0 disease. **(a)** At the 3-month post-treatment PET-CT, the patient presented with a left-sided, moderate focal vocal cord uptake (SUV_max_ 3.7) with expansion, left vocal cord paresis, opposite side arytenoid region uptake, and anterior neck muscle uptake. A biopsy of the vocal cord showed mild chronic inflammation and scar tissue, consistent with post-radiotherapy changes. The patient developed a strong supraglottic oedema, pain, and hoarseness. **(b)** In a repeat PET-CT 6 months later, the uptake area had progressed in size and intensity (SUV_max_ 7.0). The PET-CT revealed solitary lung metastasis (not shown). The residual tumor was surgically treated, but the patient later succumbed to the metastatic disease

The three patients in our cohort who had a positive PET-CT and negative biopsy, and local recurrence detected at 7, 9, and 11 months, may have harbored an active malignant process at the time of the first imaging. The SUV_max_ of the primary tumor area were 3.7, 4.7, and 4.2, respectively, and all had a persistent mass on the CT. The diagnosis of recurrence may have been delayed 5 months, 3.5 months, and 8 months, respectively, because of the initial false negative biopsy (Fig. 3). According to our criteria for residual disease with the positive biopsy result before 6 months as the gold standard, these patients were classified as non-residuals. If they are defined as residuals, our interpretation specificity increases to 78.7% (from 75.0%), and PPV increases to 48.0% (from 36.0%). The false negative biopsy rate was 23.1% (3 out of 13 negative biopsies taken before 6 months from the end of treatment).

One patient had negative initial PET-CT at 3 months post-treatment but was diagnosed with a recurrence at primary tumor site 6 months later. Suspicion of the recurrence arose at routine clinical control with subsequent positive PET-CT and biopsies. All other local recurrences occurred clearly later (over 1 year from the end of treatment).

Vocal cord uptake in non-residuals was found in 32 out of 58 (55.2%). Of these, 7 (12.1%; 7/58) were on the area of no previous tumor growth, and 25 (43.1%; 25/58) on the area of previous tumor growth. Most of these were asymmetric (90.6%), and associated with a false positive PET-CT interpretation (p = 0.001). Arytenoid cartilage area uptake was common in both groups (residuals and non-residuals), and not associated with residual tumors or false positive interpretations. In patients with no residual disease and no previous tumor growth on the arytenoid uptake area, bilateral uptake was present in 31 (53.4%; 31/58) and unilateral uptake in 6 (10.3%; 6/58). Patients with broad, symmetric, artifact-like uptake were removed from this analysis.

## Discussion

In our study, consisting of only laryngeal carcinoma patients, the NPV of PET-CT at the primary tumor area was extremely high. Thus, all patients presenting without a persistent mass, and either a minimal uptake or no uptake, had a complete local response. In assessing residual disease, however, equivocal and positive PET-CT findings are less reliable; the positive predictive value was the highest (only 46%) with SUV_max_ over 4.0 and a persistent mass present at the primary tumor area. Our study revealed better sensitivity and NPV compared to the results of a recent review consisting of 3 studies comprising mostly laryngeal carcinoma patients [5]. Another study by Slevin et al. including 35 laryngeal and hypopharyngeal carcinoma patients showed results more similar to ours: sensitivity, 100%; specificity, 73%; PPV, 46%; and NPV, 100% [12]. They reported a true positive rate of 29% in the equivocal interpretation category and a 67% true positive rate in the positive interpretation category, whereas in our series, the true positive rate was similar across these two categories. Since residual disease was almost as common in both categories, equivocal interpretation must be taken seriously when planning further diagnostics and follow-up. Reporting PET-CT findings using NI-RADS improved PPV and decreased NPV slightly, but had similar challenges as our 3-step scale reporting; mainly the difficulty of deciding between mild to moderate focal uptake and intense focal uptake, as no fixed SUV_max_ thresholds are used.

Many different SUV_max_ cut-off points have been used in previous studies for post-treatment HNC, but the consensus is lacking, probably because malignant and benign findings considerably overlap [5, 13, 14]. SUV_max_ measures metabolic rate, and it may be high in various inflammatory conditions, and low in malignant tumors that are small, and have a low metabolic rate, or because of the patient’s hyperglycemia. Murakami et al. showed SUV_max_ over 3.0 to be a usable threshold in malignant neck nodes over 15 mm in diameter [15], and we used it to differentiate abnormal uptake. In our cohort, all local residuals had SUV_max_ over 4.0, but 28% of the non-residuals also surpassed this threshold. In a study by Slevin et al., all local laryngeal residual tumors that had SUV_max_ reported, showed SUV_max_ over 4.0 [12]. Oe et al. used SUV_max_ cut-off value of 3.35 to achieve a sensitivity estimate of 93.75% and a specificity estimate of 91.67%, but the results are not completely comparable to ours as the primary treatment modalities included surgery [16]. In another investigation comprising laryngeal carcinoma patients, the post-treatment SUV_max_ for individual imaging studies were not specified or it remained unclear whether the tumor was residual or recurrent [17, 18]. With only 9 local residual tumors, it is too early to suggest changes in the interpretation criteria based on our study alone.

**Fig. 4:**
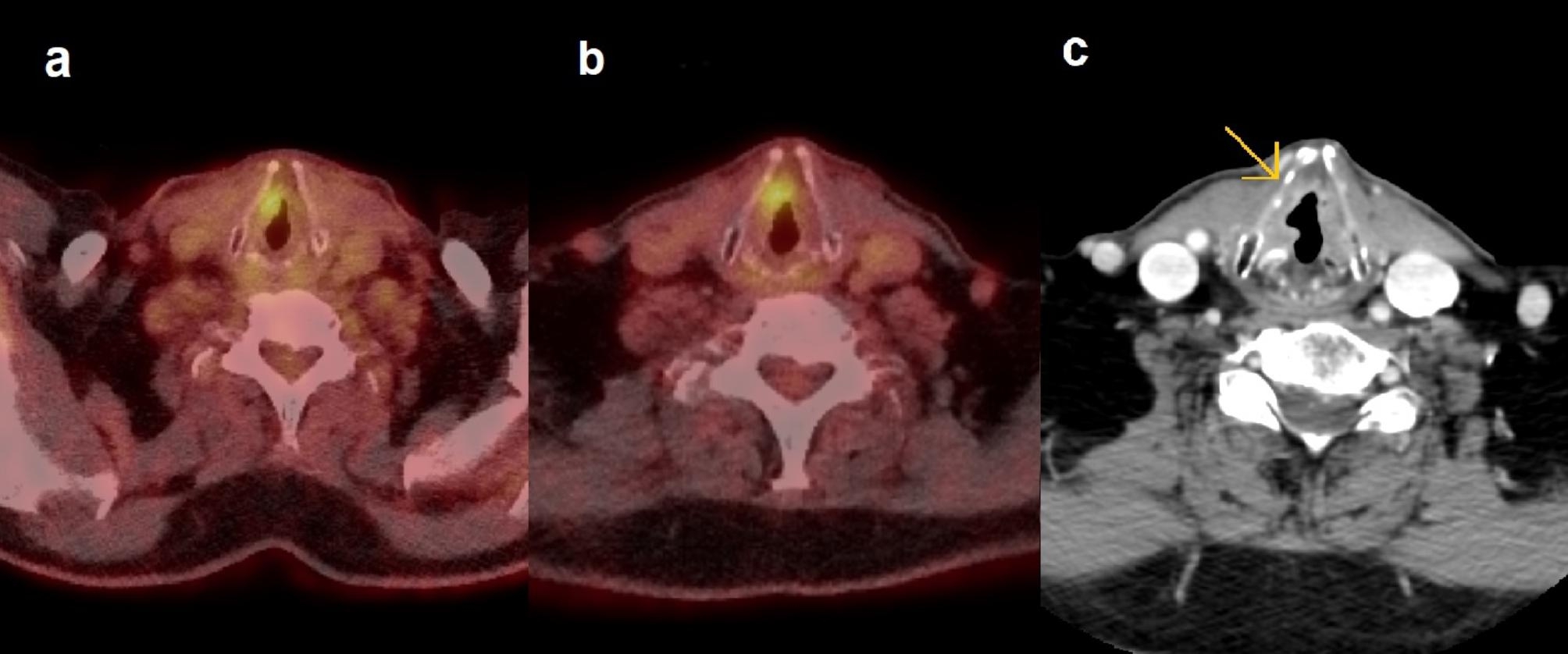
False positive result on two consecutive PET-CTs. On the PET-CT 3 months after the end of treatment, the area of the previous primary tumor around the right laryngeal ventricle showed a focal mild radiotracer uptake of SUV_max_ 3.2 **(a)**. The laryngoscopy revealed a small bump on the anterior vocal cord, which revealed only mild chronic inflammation on biopsy. The PET-CT was repeated 9 months later because of hoarseness and an infectious-looking lesion on the false cord. Focal uptake on the previously detected uptake area had increased to 4.1 **(b)**. On laryngoscopy, the false cord appeared exophytic, and biopsies showed epithelial hyperkeratosis and hyperplasia. Two and half years later (three and half years after end of treatment), the patient returned with a right-sided glottic tumor growth with biopsies resulting in SCC. CT **(c)** showed a malignant-looking enhancing lesion with ulceration on the anterior commissure, and anterior and middle thirds of the vocal cord

**Fig. 5:**
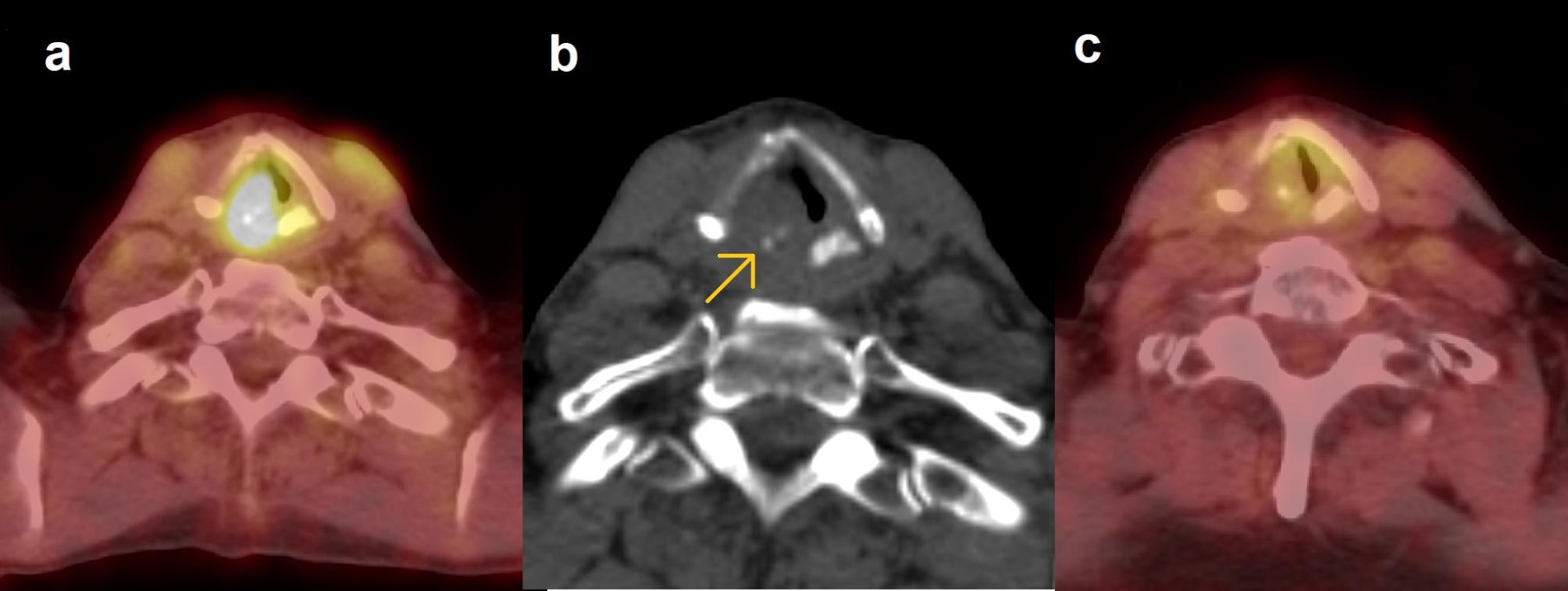
Example of a false positive interpretation in the case of cricoid cartilage radionecrosis in a T4aN0 tumor. **(a)** Post-treatment PET-CT showed a markedly increased uptake of SUV_max_ 8.3 at the right posterior vocal cord and arytenoid region, along with a mass, at the site of the primary tumor. The axial image **(b)** shows small, fragmented arytenoid cartilage (arrow). A subsequent laryngoscopy revealed defects in the mucosa and exposed cartilage. The biopsy was negative for residual disease. The patient developed difficulty in breathing and a laryngeal oedema, and was tracheostomized. Symptoms resolved after antibiotics and hyperbaric oxygen therapy, which were started due to suspected chondroradionecrosis. **(c)** In the follow-up PET-CT 8 months later, SUV_max_ had markedly decreased to 3.6

Abnormal vocal cord uptake in the previous primary tumor area was present in a substantial proportion of non-residuals (43%), and SUV_max_ values overlapped with those with residual tumors, which understandably was associated with false positive interpretations (Fig. 4). Arytenoid area uptake was common and it was not associated with residual tumors. Post-treatment changes as well as talking or coughing after the radiotracer injection, often result in an increased uptake in vocal cords or in the arytenoid region, the posterior cricoarytenoid muscles, or dorsally in the pharyngeal constrictor muscles. The uptake was occasionally unilateral, which can also point to post-treatment mucosal ulceration, contralateral compensatory muscle activation in vocal cord paralysis, or cartilage necrosis [19, 20] (Fig. 5).

Although the mass on CT is used in NI-RADS classification, we could not find studies that examine this imaging marker’s effectiveness in post-treatment laryngeal carcinoma. We hypothesized that it would help in differentiating residuals from post-treatment changes. The mass alone proved to be more common in residuals but also occurred relatively often in non-residuals. When SUV_max_ over 4.0 and mass were combined, specificity and NPV were high, so this combination usually indicated the presence of residuals. The absence of mass, however, could not rule out residual disease. Three patients in our cohort exhibited elevated SUV_max_, persistent mass, and positive PET-CT interpretation, but biopsies were negative. All had local recurrence soon after. In this kind of situation, negative biopsy results should be regarded with suspicion.

A direct laryngoscopy of a suspected residual tumor requires general anesthesia and does not always diagnose the residual tumor correctly. Biopsies may complicate healing and make the interpretation of subsequent imaging more difficult. Comparatively, in a study with 131 radiotherapy-treated laryngeal carcinomas, the false negative rate regarding biopsies of suspected recurrence was 31% [4]. Repeated imaging after an equivocal result may reduce unnecessary laryngoscopies. For most patients presenting with a local residual tumor after (C)RT for T3-4 laryngeal cancer, the only option for local clearance is total laryngectomy. Although residual tumors should preferably be detected without delay, the “wait and re-scan” policy for up to 4 to 6 months after primary treatment is not likely to affect the outcome [21].

Two studies including various HNCs, laryngeal cancers being the minority, found out that in the repeated PET-CT one month or later after the initial post-treatment equivocal PET-CT, the conversion to a complete response rate was 48% [22] and 60% [23]. Likewise, in a smaller series consisting mostly of laryngeal carcinoma patients, 9 out of 12 (75%) patients with a positive PET-CT and negative biopsy had a decreased FDG uptake in the repeat scan [9]. An additional imaging causing ionizing radiation might be justified in these RT-treated patients to reduce false positive findings, and to avoid unnecessary laryngoscopies.

MRI that includes diffusion weighted imaging (MR-DWI) has also shown promise in differentiating laryngeal or hypopharyngeal residual tumors, with one series achieving a sensitivity of 94% and a specificity of 100% [24]. We could not find studies that compare MRI and PET-CT specifically in post-treatment laryngeal carcinoma. Various post-treatment changes, as well as physiological phenomena like talking that cause false positive results in the PET-CT, usually do not cause interpretation problems in the MRI-DWI. Motion artifacts, however, to which the DWI sequence is sensitive to, may make it difficult to evaluate small residual tumors. Also, as a standalone study, the head and neck MRI lacks the whole-body data and the opportunity to rule out distant metastases. A combined PET-MRI is increasingly available in many clinical centers, and preliminary studies on all HNCs show great performance on recurrence detection [25].

In our univariate analysis of pre-treatment prognostic factors, we found that a larger primary tumor diameter and vocal cord fixation were positively correlated with local residual or recurrent disease. Primary tumor size, measured as volume, was found to have strong prognostic value in a recent systematic review and meta-analysis examining imaging factors associated with laryngeal or hypopharyngeal carcinoma recurrence after CRT [26]. This finding is interesting because primary tumor size is not included in TNM classification [27], but in our cohort, it correlated positively with T-stages 3 and 4 (versus T-stage 2). Vocal cord fixation is one of the criteria for T-stage 3. Predictably, in multivariable analysis adjusted for T-stages 3–4, the correlation for any of these factors disappeared. It is possible that a larger study population is needed to perform robust multivariable analysis.

Our study has the advantage of including over twice the number of patients compared with previous studies including only laryngeal carcinoma patients [17, 18]. The number of local residuals, however, was relatively low, which makes it harder to perform a reliable statistical analysis. Laryngeal carcinomas are relatively rare and despite a twelve-year period of collection at our tertiary care hospital, no larger cohort could be obtained. The post-treatment PET-CT timing was somewhat heterogeneous even though the protocol states that the optimal imaging time is 3 months. This spread in imaging timing reflects reality in patient care. We excluded PET-CTs performed earlier than 2 months after treatment to minimize false positive results that span from imaging that occurred too early.

Defining a time threshold for residual disease too close to the post-treatment imaging may lead to a false negative scan to be interpreted as a true negative, if biopsy confirmation of a relapse is done after the residual time threshold has passed. This would potentially increase the sensitivity of the imaging test. In our cohort there was one patient with early recurrence and with negative PET-CT. The routine post-treatment PET-CT was performed 3 months after treatment and the suspicion of the recurrence arose 6 months later, confirmed by focal strong uptake at PET-CT, and positive biopsy. Biopsy was not taken after the initial negative scan so it cannot be known for sure if this was indeed a residual, but the time interval between the scan and the recurrence diagnosis seems sufficient to condemn this event as a recurrence and not a residual. Negative PET-CTs without a clinical suspicion are not confirmed with biopsies, which leads to a phenomenon called partial confirmation bias. This potentially could increase sensitivity and decrease specificity. It is a common problem in diagnostic test study designs.

Repeated imaging with PET-CT or MR-DWI instead of laryngoscopy and biopsies in equivocal and positive cases could be a feasible future research topic. Also, the ability of a combined PET-MRI scan to find recurrence, specifically in laryngeal cancer, remains to be explored. Another worthwhile topic is to investigate in a large patient cohort whether delaying the first post-treatment imaging to 4 or 5 months would produce more accurate results, and help to reduce false positive interpretations.

## Conclusion

In our study, the negative interpretation was reliable, but equivocal and positive results have low PPV and require further diagnostics. Local residual was as common in the equivocal interpretation category as in the positive interpretation category. Interpretation is complicated by overlapping SUV_max_ values and common focal uptake in the primary tumor area in non-residuals. All residuals had SUV_max_ over 4.0. Combining this factor with a presence of mass in the CT increased specificity, but this combination could not be used as the sole deciding factor, as its sensitivity was low.

## Data Availability

The datasets used and/or analyzed during the current study are available from the corresponding author on reasonable request.

## Abbreviations

18-F-FDG: 18-Fluoride-fluorodeoxyglucose
CRT: Chemoradiotherapy
CT: Computed tomography
DSS: Disease specific survival
HNC: Head and neck cancer
LPR: Larynx preservation rate
MRI: Magnetic resonance imaging
MRI-DWI: Magnetic resonance imaging with diffusion weighted imaging
NI-RADS: Neck Imaging Reporting and Data System
NPV: Negative predictive value
PET-CT: Positron emission tomography and computed tomography
PET-MRI: Positron emission tomography and magnetic resonance imaging
PPV: Positive predictive value
ROI: Region of interest
RT: Radiotherapy
SCC: Squamous cell carcinoma
SUV_max_: Maximum standard uptake value
